# Supplementary figures and images for: Spatial repellents transfluthrin and metofluthrin affect the behavior of Dermacentor variabilis, Amblyomma americanum, and Ixodes scapularis in an in vitro vertical climb assay
Source: PLoS One. 2022 Nov 8;17(11):e0269150. doi: 10.1371/journal.pone.0269150 (PMC9642883; doi:10.1371/journal.pone.0269150)

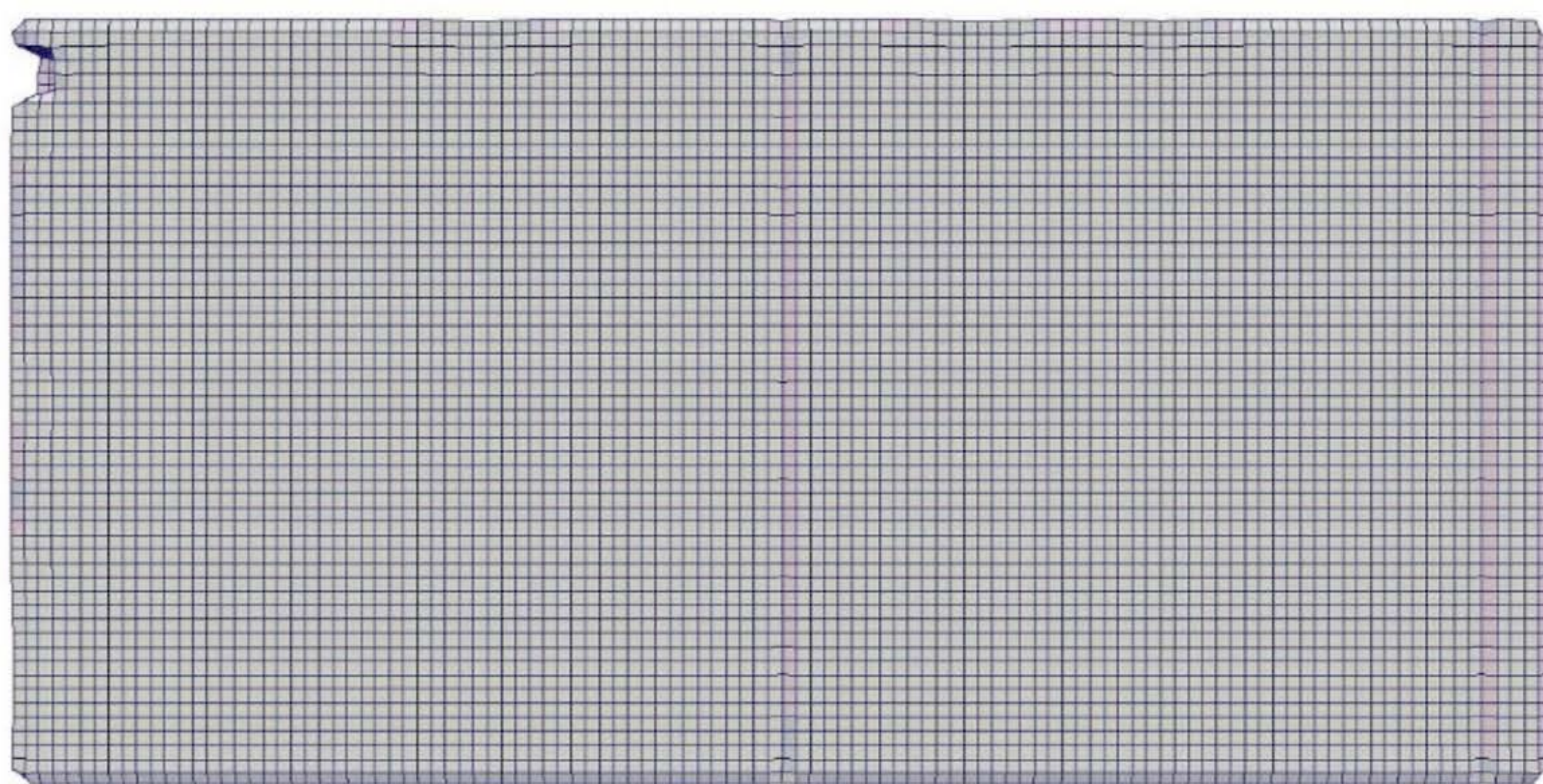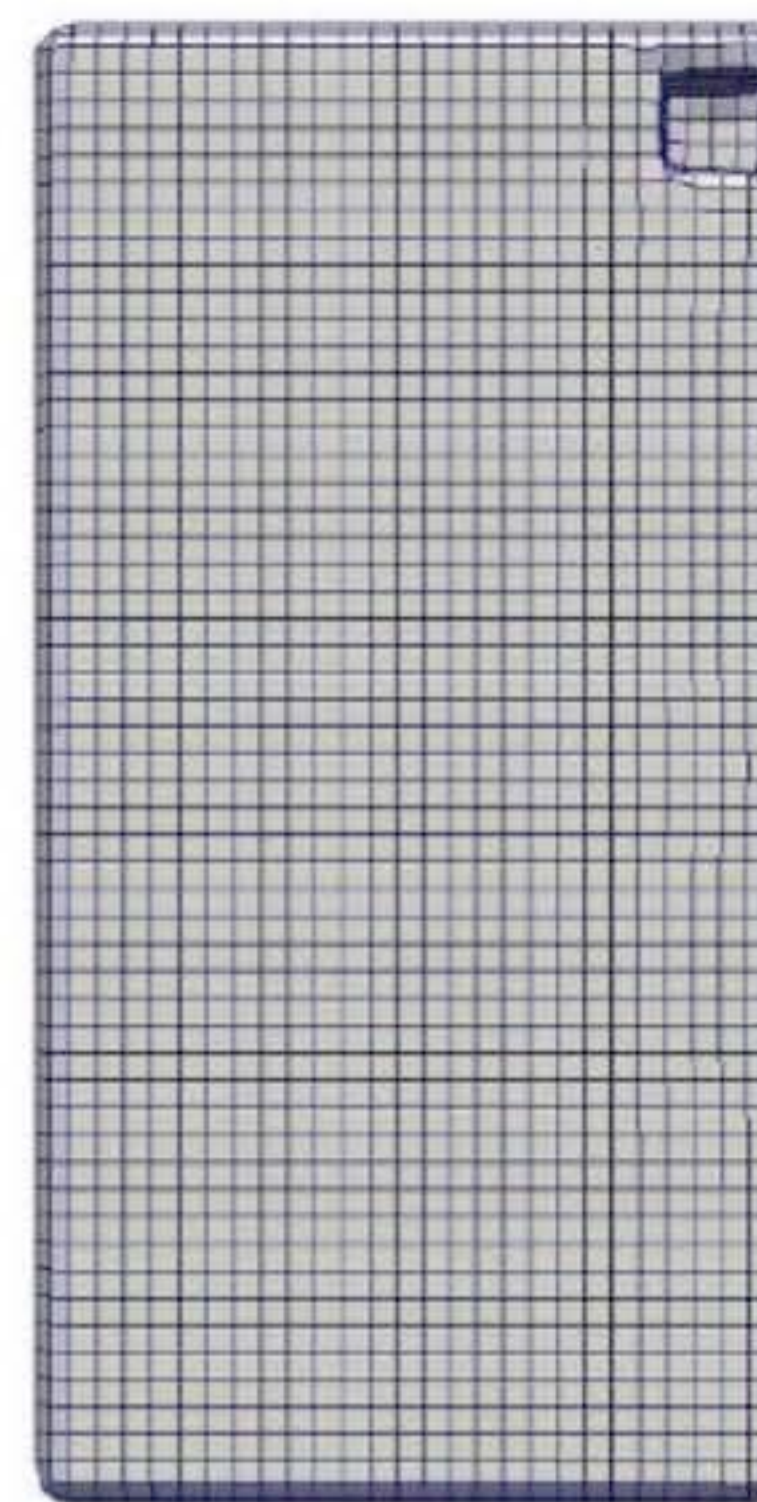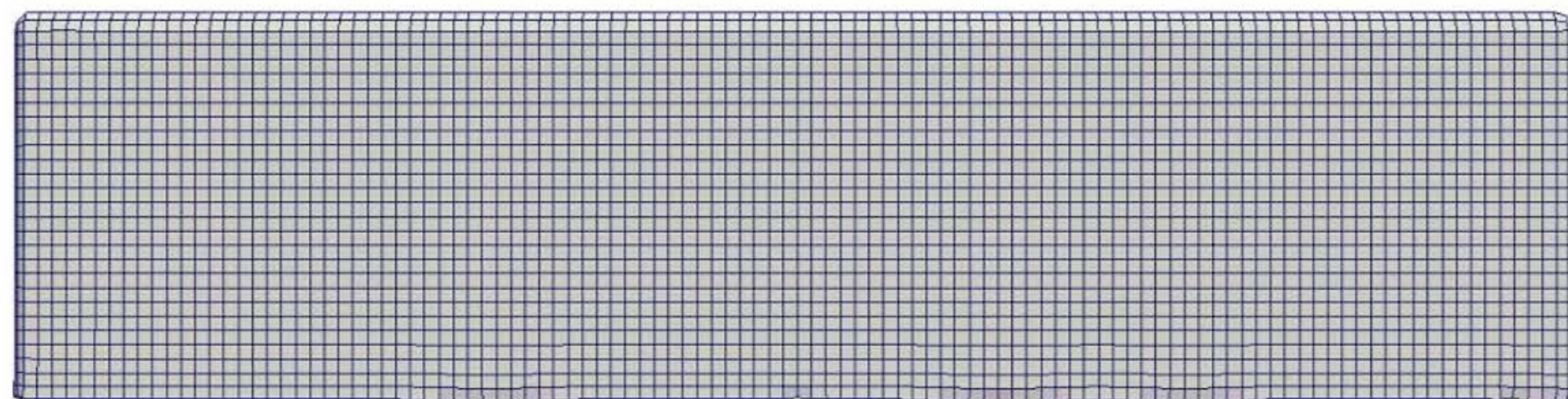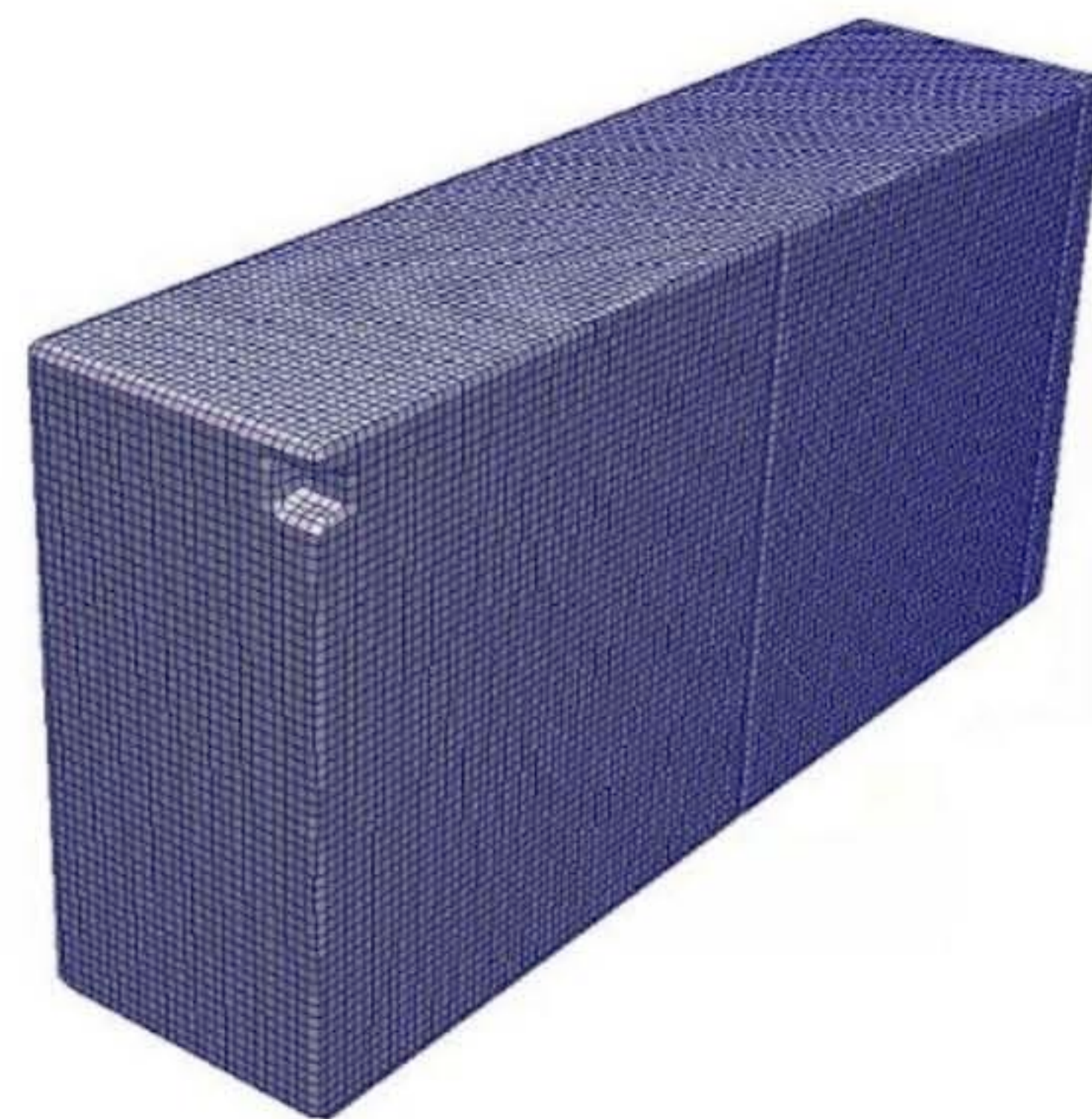

Supplement: S1 Fig — (PDF) [file pone.0269150.s001.pdf]

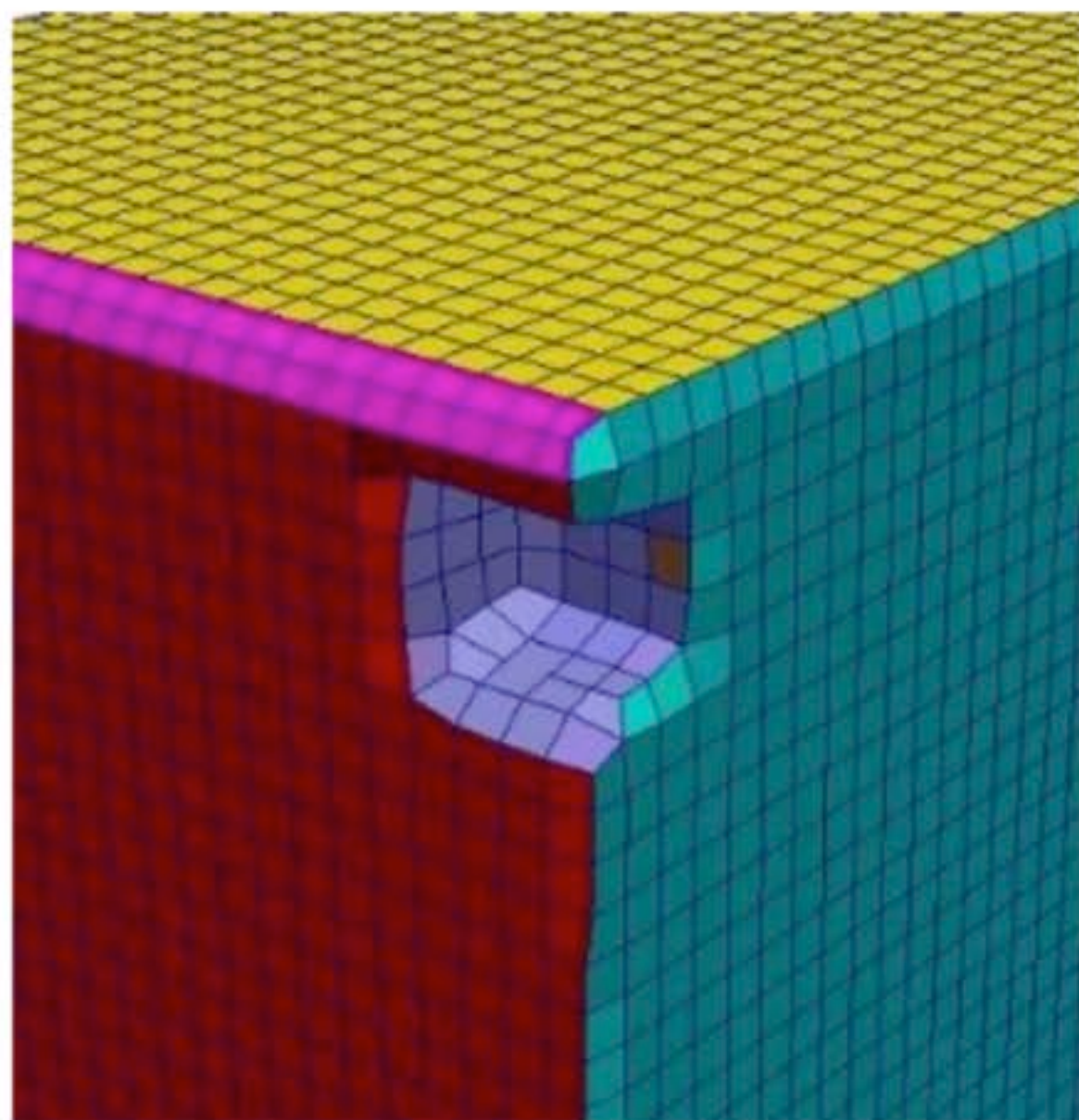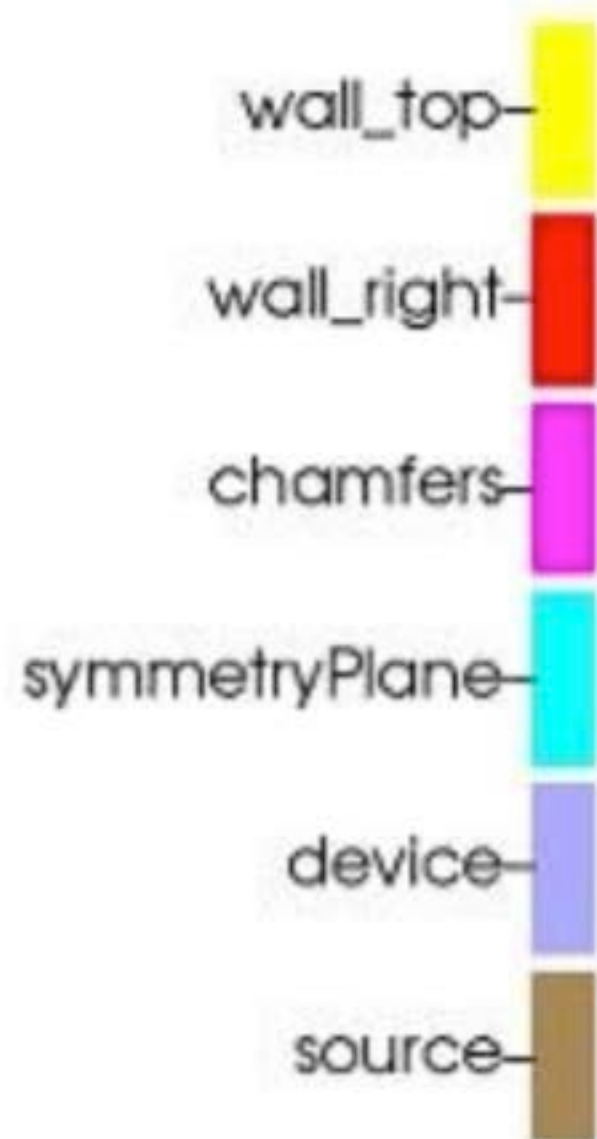

Supplement: S2 Fig — (PDF) [file pone.0269150.s002.pdf]
