# Supplementary material for: Spatial repellents transfluthrin and metofluthrin affect the behavior of Dermacentor variabilis, Amblyomma americanum, and Ixodes scapularis in an in vitro vertical climb assay
Source: PLoS One. 2022 Nov 8;17(11):e0269150. doi: 10.1371/journal.pone.0269150 (PMC9642883; doi:10.1371/journal.pone.0269150)
Supplement: S1 Table — (PDF) [file pone.0269150.s003.pdf]

Fluid	Molar mass [g/mol]	Diffusivity in air [m^2/s]	Mass flow rate [ug/hr]	Molar inlet fraction	Ideal gas density [kg/m3]
Transfluthrin	371.15	4.27e-6	3100	8.8823e-9	15.1789
Metofluthrin	360.34	4.1 le-6	4400	1.93437e-8	14.7368
Isopropanol	60.1	1.00162e-5	27430	0.053	2.4579
