# Supplementary material for: Spatial repellents transfluthrin and metofluthrin affect the behavior of Dermacentor variabilis, Amblyomma americanum, and Ixodes scapularis in an in vitro vertical climb assay
Source: PLoS One. 2022 Nov 8;17(11):e0269150. doi: 10.1371/journal.pone.0269150 (PMC9642883; doi:10.1371/journal.pone.0269150)
Supplement: S1 Appendix — (DOCX) [file pone.0269150.s004.docx]

**Appendix**

**A1. In Silico Experiments**

**A1.1 Coupled General Solver**

To model mass transport phenomena of the formulated spatial repellents in air, a coupled solution was developed within OpenFOAM combining multiphase EulerFoam and InterFoam solvers in order to simultaneously solve the combined convection and diffusion of three components: air, isopropyl alcohol and the spatial repellent [75-78]. In this model the mixture was treated as a single fluid with variable density according to its composition. The model incorporated the three linear momentum equations coupled with the mass continuity equation to obtain mean velocity distributions for the mixture and the three component volume fractions.

For each component the mass continuity equation for each component is stated in the following general form for n-components:

$\frac{d\alpha_{i}}{dt}+\nabla\cdot\left( \bar{U}\alpha_{i} \right)-\sum_{j=1}^{n} \nabla\cdot\left( D_{ij}{\nabla\alpha}_{i} \right)=0 in \Omega,$(1)

where i is the index representation for each component (e.g. 1 = air, 2 = spatial repellent, 3 = isopropanol), $\alpha_{i}$ is the volume fraction of the i-th component, $\bar{U}$ is the fluid velocity and $D_{ij}$ is the diffusion coefficient between the i-th and j-th component and $\Omega$ the volume of the chamber. The first term is the time derivative, the second term the convective derivative and the last term addresses the diffusion of the i-th component into the other components. In this model, we consider that   $D_{ij}$=$D_{ji}$ following Chapman–Enskog theory.

The three momentum equations are:

$\frac{\partial\left( \rho\bar{U} \right)}{\partial t}+\nabla\cdot\left( \rho\bar{U}\bar{U} \right)+\nabla p-\nabla\tau-\rho\bar{g}=0 in \Omega,$ (2)

where $\bar{U}$ is the fluid velocity,  $\rho$ the fluid mixture density, $p$ the pressure,  $\bar{g}$ the gravity and  $\tau$ the fluid deviatoric stress tensor and $\Omega$ the volume of the chamber. The stress tensor is obtained from Random Averaged Navier Stokes equations where turbulence is addressed through the SST k-ω model [79-80].

Since the momentum equations are solved for the mixture and not for each component, diffusion between components is not considered in this equation. Alternatively, diffusion effects affect the fluid flow through the density field.

For each time step, the continuity equations (1) were first solved for each single volume fraction component. Second, the resulting fluid mixture density was calculated using each individual component densities and volume fractions with the following equation:

$\rho=\sum_{i=1}^{n} {\rho_{i}\alpha}_{i}=0 in \Omega,$ (3)

Finally, $\rho$ is plugged into the momentum conservation equation (2) and the procedure is repeated until convergence is achieved.

To implement equations (1-3) in the selected software simulation package (OpenFOAM), equation (1) is rearranged to adapt it to a flux-based representation, as follows:

$\frac{d\alpha_{i}}{dt}+\nabla\cdot\left( \bar{U}\alpha_{i}-\sum_{j=1}^{n} D_{ij}{\nabla\alpha}_{i} \right)=0 in \Omega,$(4)

which is of the form:

$\frac{d\alpha_{i}}{dt}+\nabla\cdot F_{i}=0$, (5)

where $F_{i}$ is the flux of $\alpha_{i}$. This equation was solved with the available Flux-Corrected-Transport (FCT) algorithm called Multidimensional Universal Limiter for Explicit Solution (MULES), which in turn was used to solve the momentum equations (2) using the PIMPLE algorithm.

**A1.2 Boundary Conditions**

In the in silico model the release of the formulated spatial repellent is modeled as two separate components: the spatial repellents and the isopropanol. The evaporation rate of the formulated spatial repellent was experimentally measured and calculated as two separate release rates for the spatial repellent and the isopropanol. These rates were integrated into the model as two separate sources that release mass into the chamber. For the gaps found at the chamber vertices due to the assembly of the acrylic panels, an advective boundary condition for the velocity was defined at these edges to allow outward fluid mixture flow, while keeping the pressure constant at atmospheric pressure.

As both the geometry and the boundary conditions are symmetrical along the longitudinal plane, only half of the problem was simulated and solved to reduce the computational cost. No slip wall conditions were applied at the chamber walls and CRD outer structure.

**A1.3 Mesh Details**

A regular hexahedral mesh was used. S1 Fig shows the geometry and general mesh and S2 Fig shows a detailed view of the release source for both spatial repellent and isopropanol, chamfers and device mesh for the simulation. The test bench was accurately meshed to account for the CRD, the sticks, and the chamber walls. For the source, an area was identified to release the respective spatial repellent and isopropanol.

**A1.4 Component Inlet Rates and Physical Properties Determination**

Due to the expected low concentration of the formulated AIs, transfluthrin and metofluthrin in vapor phase were treated as ideal gases. The ideal gas density that transfluthrin, metofluthrin and isopropanol would have at the reference temperature (25°C) and pressure (1 atm) using the ideal gas law was determined from:

$\rho_{i}=\frac{p_{ref}}{R}\frac{M_{i}}{T_{ref}}$,        (6)

where $R$ is the universal gas constant (8.314 $\frac{J}{mol K}$ ) M is the molar mass, T is the temperature and p is the pressure.  This ideal gas density was never reached during simulations but required for model development and is only valid for low concentration calculations. The air density was taken to be $\rho_{air}$=1.205$\frac{kg}{m^{3}}$. The fluid properties of transfluthrin, metofluthrin and isopropanol, as well as their measured evaporation rates, are summarized in S1 Table. The diffusion between spatial repellents and isopropanol was assumed to be negligible.

**References**

1. Weller HG, Tabor G, Hasak H, Fureby C. A tensorial approach to computational continuum mechanics using object-oriented techniques. Computers in Physics. 1998;12(6). doi: 10.1063/1.168744.
2. Deshpande SS, Anumolu L, Trujillo MF. Evaluating the performance of the two phase flow solver interFoam. Computational Science & Discovery. 2012; 5:14-16.
3. Wardle KE, Weller HG. Hybrid multiphase CFD solver for coupled dispersed/segregated flows in liquid-liquid extraction. International Journal of Chemical Engineering. 2013
4. OpenFOAM: OpenCFD Ltd, Bracknell, United Kingdom. Available from: htpps://www.openfoam.com, 2021.
5. Menter FR. Two equation eddy-viscosity turbulence modeling for engineering applications. AIAA Journal. 1994;32: 1598-1605. doi: 10.2514/3.12149.
6. Greenshields C. OpenFOAM 2.3.0: Multiphase modeling predictor-corrector semi-implicit MULES.
