# Supplementary material for: Spatial repellents transfluthrin and metofluthrin affect the behavior of Dermacentor variabilis, Amblyomma americanum, and Ixodes scapularis in an in vitro vertical climb assay
Source: PLoS One. 2022 Nov 8;17(11):e0269150. doi: 10.1371/journal.pone.0269150 (PMC9642883; doi:10.1371/journal.pone.0269150)
Supplement: Attachment — Submitted filename: response to reviewers.docx [file pone.0269150.s008.docx]

General comments: Spatial repellents transfluthrin and metofluthrin affect the behavior of *Dermacentor*

*Variabilis,* *Amblyomma americanum*, and *Ixodes scapularis* in an

*in vitro* vertical climb assay

The authors thank the reviewer for the feedback. All individual points raised have been addressed as noted below:

The manuscript is a well-detailed investigation of a relatively low-complexity test method and parameters for scoring when using semi-volatile active ingredients against ticks. I do sort of wonder at the labor-requirement in using videography for scoring, but for the most part I think this is a very accessible testing architecture for other research groups. I was not part of the first review cycle, so, as my first review of this work, I feel that there is a lot of written polish and good logic presented throughout. I have only minor suggestions to help strengthen the paper. Otherwise, I believe this is an excellent contribution to tick research and spatial repellent methodology.

Abstract:

My personal preference is to be a bit more specific in the abstract. These are minor suggestions:

1. Line 38 – What are these parameters?

The authors thank the reviewer for the feedback regarding the abstract structure. Please refer to lines 39-40 on the updated manuscript, where the parameters are now defined.

2. Line 39-41 – To what extent? Can you give something a bit more concrete and quantitative, like a % of ticks that refused to cross a certain distance into the plume or something?

The authors thank the reviewer for this additional suggestion in the abstract and agree that this addition strengthens it. Please refer to lines 42-46, where the parameter which we feel is most important/representative of the study goals has been quantified to give the readers a better idea of the effect of spatial repellents in this study.

Introduction:

3. Line 57 – Could you be more specific? Relay to what extent incidence has risen (such as a range of case increases or something). Dramatically isn’t very easy to assign a value to.

The authors thank the reviewer for raising this point. We agree that adding this information is useful to the readers. Please refer to lines 56-59 in the updated introduction, where this is now revised.

4. Line 64 – I would argue that personal protection is the only true pillar of tick bite prevention, as there are no meaningful trapping methods (tick drags are very labor intensive and inconsistent) and chemical control is laughable (routinely dousing leaf litter with broad spectrum insecticides is neither targeted nor very effective; only desperate).

The authors thank the reviewer for this comment. The language used in line 65 has been revised to better represent the role that personal protection plays in tick bite protection.

The rest of the introduction is very comprehensive and well written. I like the articulated argument made about redefining repellency standards for ticks and the logic given for why that matters.

Materials and Methods:

5. Line 114-115 – Please indicate the purity of your starting material (ex: 94% technical grade). Also, generic supplier is not very informative; Sumitomo still controls the majority of product flow for metofluthrin. There are varying isomeric purities that matter for all pyrethroids, including the penchant for R-isomerization that even the polyfluorinated structures exhibit. I think you should be more specific about the supplier. If it is in-house or proprietary, that is important to be aware of as well.

The authors appreciate the feedback regarding the materials section. The sourcing, technical grade, and isomeric composition of the active ingredients has been revised where needed. This can be found on lines 114-117.

6. Line 126 – 127 – I assume you were maintaining a chill coma? You should specify if you were clearly inducing chill coma because there are also more efforts recently that study the cold-hardening processes in ticks, which lowers the chill coma temp at the expense of resilience.

The authors thank the reviewer for raising this point of clarity in the materials and methods. The storage and handling information has been revised to clarify that a chilled coma was maintained until the ticks were used. This can be found on line 128.

7. Line 149 – So this was not a ventilated box? Please clearly indicate if this was a static air apparatus, particular since there is some division in literature between studies doing static air olfactometry versus filamentous/laminarized plume-airflow systems.

The authors thank the reviewer for raising this additional point of clarity in the materials and methods. Please refer to lines 151-152, where it has been clarified that ventilation occurs between the outside air and the air in the chamber.

8. Line 165 – Please reiterate here your dilution to 30%; it’s easy to miss your one mention of it on Line 116.

The authors agree with the reviewer that this should be restated. Please find this done on like 168.

Results/Discussion:

The authors did an excellent job of bridging their investigations to the intended root of application in most ways (good bioassays, good exposure paradigms, good bridging with IPM practices).

I don’t have much to argue with in the bulk of their points as they are well formulated thoughts with a plethora of literature. I will point out just a couple “watch outs.”

9. Line 502 – 505 – I would counter that the main limiter to spatial repellents against ticks is the poor integrity of the gradient. Either because of increased vapor pressure resulting from humidity or losing the envelope of protection to air movement/actively walking out of the vapor. Therefore, I personally think there is much greater risk the CRD will not protect a large enough area to prevent bites. You hint at this to some degree with your data showing a very visible loss in magnitude of concentration within only 20 cm.

The authors thank the reviewer for the feedback in the discussion section. Lines 501-504 have been revised to include changes in vapor pressure and stress that the integrity of the gradient barrier, which serves as the basis of protection with CRDs, may be affected with certain external factors.
